# A vaccine consisting of *Schistosoma mansoni* cathepsin B formulated in Montanide ISA 720 VG induces high level protection against murine schistosomiasis

**DOI:** 10.1186/s12879-016-1444-z

**Published:** 2016-03-05

**Authors:** Alessandra Ricciardi, Kittipos Visitsunthorn, John P. Dalton, Momar Ndao

**Affiliations:** Department of Microbiology & Immunology, McGill University, Montreal, QC Canada; Institute of Parasitology, McGill University, Montreal, QC Canada; School of Biological Sciences, Medical Biology Centre (MBC) Queen’s University Belfast, Belfast, Northern Ireland; National Reference Center for Parasitology, Research Institute of the McGill University Health Center, Montreal, QC Canada

**Keywords:** Schistosomiasis, Vaccine, Cathepsin B, Montanide ISA 720 VG, Antigen-specific antibodies, Cytokine production

## Abstract

**Background:**

Schistosomiasis is the most important human helminth infection due to its impact on public health. The clinical manifestations are chronic and significantly decrease an individual’s quality of life. Infected individuals suffer from long-term organ pathologies including fibrosis which eventually leads to organ failure. The development of a vaccine against this parasitic disease would contribute to a long-lasting decrease in disease spectrum and transmission.

**Method:**

Our group has chosen *Schistosoma mansoni* (Sm) cathepsin B, a peptidase involved in parasite feeding, as a prospective vaccine candidate. Our experimental formulation consisted of recombinant Sm-cathepsin B formulated in Montanide ISA 720 VG, a squalene based adjuvant containing a mannide mono-oleate emulsifier. Parasitological burden was assessed by determining adult worm, hepatic egg, and intestinal egg numbers in each mouse. Serum was used in ELISAs to evaluate production of antigen-specific antibodies, and isolated splenocytes were stimulated with the antigen for the analysis of cytokine secretion levels.

**Results:**

The Sm-cathepsin B and Montanide formulation conferred protection against a challenge infection by significantly reducing all forms of parasitological burdens. Worm burden, hepatic egg burden and intestinal egg burden were decreased by 60 %, 62 %, and 56 %, respectively in immunized animals compared to controls (*P* = 0.0002, *P* < 0.0001, *P* = 0.0009, respectively). Immunizations with the vaccine elicited robust production of Sm-cathepsin B specific antibodies (endpoint titers = 122,880). Both antigen-specific IgG1 and IgG2c titers were observed, with the former having more elevated titers. Furthermore, splenocytes isolated from the immunized animals, compared to control animals, secreted higher levels of key Th1 cytokines, IFN-γ, IL-12, and TNF-α, as well as the Th2 cytokines IL-5 and IL-4 when stimulated with recombinant Sm-cathepsin B. The Th17 cytokine IL-17, the chemokine CCL5, and the growth factor GM-CSF were also significantly increased in the immunized animals compared to the controls.

**Conclusion:**

The formulation tested in this study was able to significantly reduce all forms of parasite burden, stimulate robust production of antigen-specific antibodies, and induce a mixed Th1/Th2 response. These results highlight the potential of Sm-cathepsin B/Montanide ISA 720 VG as a vaccine candidate against schistosomiasis.

**Electronic supplementary material:**

The online version of this article (doi:10.1186/s12879-016-1444-z) contains supplementary material, which is available to authorized users.

## Background

Schistosomiasis, caused by trematodes belonging to the *Schistosoma* genus, is an important neglected tropical disease with a substantial impact on public health. Over 200 million people are infected with approximately half of these being school aged children [1–3]. It has long been argued that the schistosomiasis disease burden is greatly underestimated, and it has been suggested that the number of infected individuals surpasses 400 million [4]. This underestimation is likely due to the inability of current diagnostic methods to detect light infections. Schistosomiasis is a chronic illness that significantly diminishes quality of life, and, in affected children, interferes with growth and cognitive development [2, 5, 6]. Treatment of schistosomiasis relies solely on the drug praziquantel which is distributed in mass drug administration control programs. However, program outcomes have been disappointing [3]. In 2011, of the 112 million children in need of praziquantel, only 16 million received treatment [7]. School-based mass drug administration programs do not take into account that as many as 40 % of children in sub-Saharan Africa are not enrolled in school [8]. It has been shown that less than 5 % of the schistosomiasis affected population is treated with praziquantel, indicating that control programs are not reaching sufficient coverage [9]. Furthermore, these mass drug administration programs are not sustainable because praziquantel does not prevent re-infection. Therefore continuous drug distribution at an optimal timing is necessary in order to maintain disease control and break the transmission cycle [8–10]. The current amount of praziquantel donated by the various sources, such as the Schistosomiasis Control Initiative, MedPharm, and Merck KgaA, is not sufficient to meet the global need [9]. Vaccines, and/or chemotherapy combined with vaccination, present the best strategy for long-term sustained control of schistosomiasis. An anti-schistosome vaccine could contribute to decreased disease spectrum and transmission by reducing worm and egg burdens. It has been stated that even a partially effective anti-schistosome vaccine could significantly help accelerate the elimination of schistosomiasis if incorporated with other control efforts such as mass drug administration, intermediate host control, and improved sanitation [11, 12].

Our group has chosen to focus on *Schistosoma mansoni* (Sm) cathepsin B as a vaccine candidate. This cysteine peptidase, originally cloned and identified by Klinkert et al., [13, 14] is predominantly found in the adult worm and migratory larva. Sm-cathepsin B is involved in the digestion of blood macromolecules. It plays a key role in hemoglobin degradation and is involved in digestion of host serum albumin or immunoglobulin G [15–19]. The physiological importance of Sm-cathepsin B has been highlighted by RNA interference (RNAi) technology which demonstrated that suppression of cathepsin B results in impeded parasite growth [20]. The present study was designed to investigate the protective potential of a Sm-cathepsin B formulation with the adjuvant Montanide ISA 720 VG (SEPPIC Inc., Fairfield, NJ, USA) in a mouse model of schistosomiasis. Montanide is a squalene based adjuvant containing a mannide mono-oleate emulsifier. The adjuvant forms water-in-oil droplets that allow for slow antigen release at the injection site. Montanide was chosen as an adjuvant because it is acceptable for use in humans; it is both safe and well-tolerated. The different Montanide adjuvants have been used in over fifty clinical trials including malaria, cancer, and human immunodeficiency virus (HIV) vaccine trials [21–23]. These adjuvants have also continuously shown to elicit robust antibody responses. In this communication, we report that an immunization regimen involving a formulation of recombinant Sm-cathepsin B and Montanide ISA 720 VG can significantly reduce worm and egg burdens.

## Methods

### Ethics statement

All animal procedures were conducted in accordance with Institutional Animal Care and Use Guidelines and were approved by the Animal Care and Use Committee at McGill University (Animal Use Protocol 7625).

### Cloning, expression, and purification of Sm-cathepsin B

The recombinant *Schistosoma mansoni* cathepsin B protein was generated as described elsewhere [24]. Briefly, *Schistosoma mansoni* cathepsin B (Genbank accession number M21309.1) cDNA was synthesized by GenScript in pUC57 with modifications in order to be suitable for expression in the yeast *Pichia pastoris* [25]. The modification process used the following primers: primer a, 5′-CAACTTGTGTGTGATTCTTTCGAAC-3′ and primer b, 5′-GTTCGAAAGAATCACACACAGGTTG-3′ [25]. Sm-cathepsin B cDNA was inserted in the pPinkα-HC vector and then transformed into yeast cells belonging to the PichiaPink™ strain 1. The recombinant Sm-cathepsin B expression was carried out following the manufacturer’s recommendations for the PichiaPink expression system (Invitrogen, Burlington, ON). Briefly, yeast cells were cultured in a flask containing buffered complex glycerol medium (1 % yeast extract [Fisher Scientific, Ottawa, ON], 2 % peptone [BD Biosciences, Mississauga, ON], 100 mM potassium phosphate pH 6.0 [Bioshop Canada Inc., Burlington, ON], 1.34 % yeast nitrogen base without amino acids with ammonium sulphate [Bioshop Canada Inc.], 0.00004 % biotin [Supelco, Bellefonte, PA], 1 % glycerol [Bioshop Canada Inc.]) at 28 °C with shaking (250 rpm) until it reached log phase growth (OD_600_ = 6). The cells were harvested by centrifugation at 3,000 × g for five minutes at room temperature. The pellet was resuspended in 200 ml induction media: buffered complex methanol medium (same recipe as BMGY except 1 % methanol [Fisher Scientific, Ottawa, ON] was used instead of glycerol). The culture was grown for three days at 28 °C with shaking (250 rpm). Every 24 h, methanol was added to a final concentration of 0.5 %. After the three days, the cells were harvested by centrifugation at 3,000 × g for five minutes at room temperature. The resulting expressed recombinant protein was purified using Ni-NTA affinity chromatography (Ni-NTA Superflow by QIAGEN, Venlo, Limburg, Netherlands). Expression quality of recombinant Sm-cathepsin B was analyzed by Coomassie staining of polyacrylamide gel and western blot using antibodies directed against the His-tag (Sigma-Aldrich, St. Louis, Mo) followed by a HRP-conjugated secondary antibody (anti-mouse IgG-HRP from Jackson Immunoresearch Laboratories, West Grove, PA).

### Immunization protocol

Female 6–8 week old C57BL/6 mice were purchased from Charles River Laboratories (Senneville, Qc). There were three immunization groups containing 10 mice each. The immunization groups were as follows: saline control mice were injected with 50 μl of phosphate buffered saline (PBS) (Wisent Bio Products, Saint-Jean-Baptiste). Adjuvant control mice received 50 μl of a 70 % volume formulation of Montanide ISA 720 VG (SEPPIC Inc., Fairfield, NJ). The experimental mice were immunized with a 50 μl solution of 20 μg recombinant Sm-cathepsin B in a 70 % volume formulation of Montanide ISA 720 VG. Each mouse was immunized intramuscularly at weeks 0, 3 and 6. The immunization timeline and the vaccine formulation (antigen: adjuvant ratio) were determined based on previous small scale immunization/challenge pilot studies (data not shown).

### *Schistosoma mansoni* challenge

*Biomphalaria glabrata* snails infected with the *Schistosoma mansoni* Puerto Rican strain were provided by the NIAID Schistosomiasis Resource Center of the Biomedical Research Institute (Rockville, MD). Cercariae, the infectious stage of the parasite, were collected from the shedding of the infected *B. glabrata* snails. Three weeks after the final immunization, all of the mice were challenged with 150 cercariae via tail exposure lasting one hour. The mice were sacrificed seven weeks post cercarial challenge. The adult worms were perfused from the hepatic portal system and counted manually [26]. The number of worms retrieved from the experimental group was compared to the control groups in order to calculate the percent reduction in worm burden. The livers and intestines of the sacrificed mice were also collected. The intestines were cut open to expose the lumen, and the contents were cleaned out by repeated rinsing with saline. The livers and intestines were then weighed and digested overnight in 4 % potassium hydroxide (KOH) [26]. The following day, the eggs present in these tissues were counted by microscopy. The number of eggs recorded from the experimental group was once again compared to the control groups in order to calculate percent reduction in egg burden. Burden reductions were calculated as follows:

$$ \begin{array}{l}\mathrm{Percent}\kern0.28em \mathrm{worms}\kern0.28em \mathrm{or}\kern0.28em \mathrm{eggs}\kern0.28em \mathrm{reduction}\\ {}=\left(1-\frac{\mathrm{Mean}\kern0.28em \mathrm{number}\kern0.28em \mathrm{of}\kern0.28em \mathrm{worms}\kern0.28em \mathrm{or}\kern0.28em \mathrm{eggs}\kern0.28em \mathrm{recovered}\kern0.28em \mathrm{in}\kern0.28em \mathrm{immunized}\kern0.28em \mathrm{mice}}{\mathrm{Mean}\kern0.28em \mathrm{number}\kern0.28em \mathrm{of}\kern0.28em \mathrm{worms}\kern0.28em \mathrm{or}\kern0.28em \mathrm{eggs}\kern0.28em \mathrm{recovered}\kern0.28em \mathrm{in}\kern0.28em \mathrm{control}\kern0.28em \mathrm{mice}}\right)\mathrm{x}\kern0.28em by\kern0.28em 100 percent\end{array} $$

### Humoral response: enzyme-linked immunosorbent assays

Blood collection via saphenous bleed was performed on each mouse prior to each immunization as well as on the day of the cercarial challenge. Sera were obtained by allowing the collected blood to clot and then removing it by centrifugation. The humoral responses elicited by the immunizations were analyzed by enzyme-linked immunosorbent assay (ELISA) as described elsewhere [24]. Briefly, 96-well plates were coated with the recombinant Sm-Cathepsin B (10 ng/well). Serial dilutions of the sera were prepared and incubated in the antigen coated plates. Sm-cathepsin B specific total IgG was analyzed as well as the isotypes IgG1 and IgG2c using secondary antibodies conjugated with horseradish peroxidase (HRP) (total IgG-HRP from Jackson Immunoresearch Laboratories, West Grove, PA; both IgG1-HRP and IgG2c-HRP from Southern Biotechnologies, Birmingham, AL). Following the addition of the substrate, 3,3′,5,5′-tetramethylbenzidine (Millipore, Billerica, MA), the plates were read at wavelength 450 nm. The samples were analysed by ELISA in duplicates and data confirmed by a single repeat. The results are expressed as mean endpoint titers (geometric mean and 95 % confidence interval). Endpoint titers refer to the reciprocal of the highest dilution that gives a reading above the cut-off [27]. Based on the number of negative controls used in the ELISAs, the cut-off was roughly equal to two standard deviations above the mean negative values [27].

### Cytokine production: multiplex ELISA

The animals were sacrificed seven weeks post cercarial challenge. The spleens of each animal were collected and splenocytes were isolated as described elsewhere [24, 28]. Briefly, the spleens were crushed and the red blood cells were lysed using ammonium-chloride potassium lysing buffer. Following repeated wash and centrifugation steps, the cells were resuspended in complete media (RPMI-1640, 10 % fetal bovine serum [Wisent Bio Products], 50 μg/ml Gentamycin, 0.05 mM 2-Mercaptoethanol). Cell counts were determined by trypan blue exclusion, and 10^6^ cells/well were seeded into 96-well plates. The cells were incubated at 37 °C in the presence of 2 μg/ml of recombinant Sm-cathepsin B. After 72 h, 100 μl of the supernatant was collected. The splenocyte supernatant was used to asses cytokine production by QUANSYS multiplex ELISA (16-plex) (Quansys Biosciences, Logan, UT) following the manufacturer’s recommendations.

### Statistical analysis

Statistical analysis was done using the Mann–Whitney test (nonparametric test) to compare different groups of data. Antibody and cytokine data are presented on semi-log plots showing geometric mean and 95 % confidence intervals. The software GraphPad Prism 5 (La Jolla, CA) was used. P values less than 0.05 were considered significant.

## Results

### Expression of recombinant Sm-cathepsin B

The expression of the recombinant Sm-cathepsin B protein used for the immunizations was shown by both Coomassie staining of polyacrylamide gel (Additional file 1: Figure S1a) and Western Blot analysis (Additional file 1: Figure S1b). By both methods, a single and clear band representing the protein can be observed at the expected 39 kDa region. Lipopolysaccharide testing revealed no detectable levels of the endotoxin.

### Protective potential of Sm-cathepsin B Formulation

**Fig. 1:**
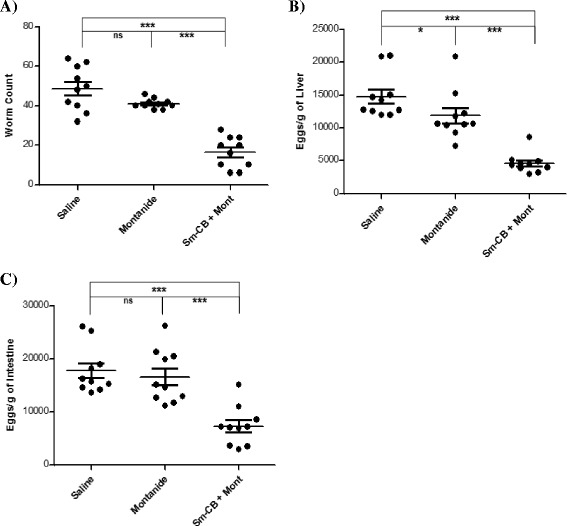
Parasitological burden. The worm counts per individual mouse (*P* = 0.0002) **a** as well as the egg load per gram of liver (*P* < 0.0001) **b** and per gram of intestine (*P* = 0.0009) **c** are represented for mice in the saline control group, the adjuvant alone control group (Montanide ISA 720VG), and the experimental group (20 μg Sm-cathepsin B with Montanide ISA 720 VG). Worm and egg burdens were determined 7 weeks post cercarial challenge. (ns = not significant, * *P* < 0.05, *** *P* < 0.001)

In order to determine the protective potential of the Sm-cathepsin B and Montanide ISA 720 VG formulation, an immunization regimen was tested using C57BL/6 mice as described above. The average worm number from the group that received the Sm-cathepsin B and Montanide ISA 720 VG formulation (16.4 ± 2.5 worms) was compared to the average from the adjuvant control group (41.1 ± 0.8 worms). Therefore, worm burden was reduced by 60.1 % in the mice that received the Sm-cathepsin B/adjuvant formulation compared to the adjuvant control mice (*P* = 0.0002) (Fig. 1a). Compared to the saline control group (48.8 ± 3.5 worms), the mice given the Sm-cathepsin B/adjuvant formulation had a 66.4 % reduction in worm burden (*P* < 0.0001). There were no statistically significant difference in worm burdens between the two control groups (*P* = 0.1285).

Schistosomiasis pathology is linked to the parasite eggs becoming trapped in tissues. Hepatic and intestinal egg burdens were determined for both the experimental and control mouse groups. Comparisons between the adjuvant control group (mean egg count = 11,876.0 ± 1,197.9 eggs/gram of liver) and the Sm-cathepsin B/adjuvant formulation group (mean egg count = 4,578.5 ± 492.9 eggs/gram of liver) revealed a 61.5 % reduction in hepatic egg burden for the mice given the vaccine (*P* < 0.0001) (Fig. 1b). When comparing the experimental group and the saline control (mean egg count = 14,791.6 ± 1,080.1 eggs/gram of liver) group, hepatic egg burden is decreased by 69.1 % in the mice that received Sm-cathepsin B/adjuvant formulation (*P* < 0.0001). There also seems to be a significant decrease in hepatic egg burden in the adjuvant control group compared to the saline group (*P* = 0.0185).

Intestinal eggs were also enumerated from the digested organ using microscopy. The group containing the animals immunized with the Sm-cathepsin B/adjuvant formulation (mean egg count = 7,292.8 ± 1,173.1 eggs/gram of intestine) had a 56.1 % reduction in intestinal egg burden compared to the group that received adjuvant only (mean egg count = 16,606.6 ± 1,590.0 eggs/gram of intestine) (*P* = 0.0009) (Fig. 1c). Compared to the saline group (mean egg count = 17,800.2 ± 1,421.8 eggs/gram of intestine), the experimental group had a 59.0 % decrease in intestinal egg burden (*P* < 0.0001). There were no statistically significant difference in egg burdens between the two control groups (*P* = 0.4813). All of the results elucidating the protective potential of the Sm-cathepsin B formulation are summarized in Additional file 2: Table S1 and Additional file 3: Table S2.

### Antibody response

**Fig. 2:**
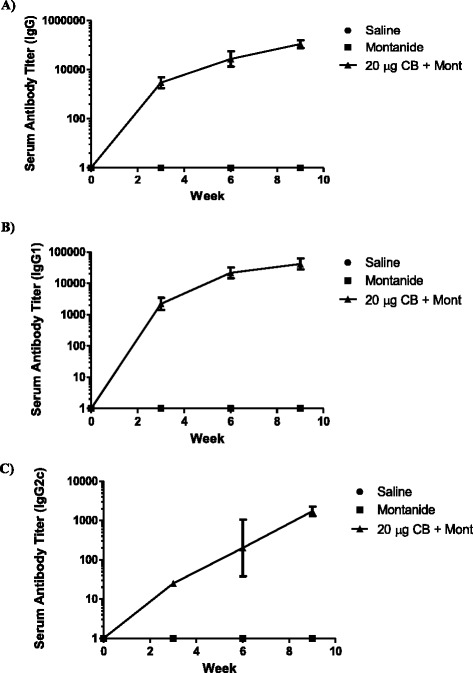
Production of Sm-Cathepsin B specific antibodies. Sm-cathepsin B specific total IgG **a**, IgG1 **b** and IgG2c **c** in immunized mice: Saline control, Montanide ISA 720 VG adjuvant control, and Sm-cathepsin B with Montanide ISA 720 VG. Serum from individual mice was analyzed by ELISA. Geometric means and 95 % confidence intervals are shown. Endpoint titers were determined for week 0, week 3, week 6, and week 9

The serum collected throughout the immunization regimen was used in order to analyze and compare the antibody production pattern between the three groups of mice used in this study. ELISAs were performed to determine antibody titers. In both the adjuvant control and saline control mice, there were no detectable levels of Sm-cathepsin B specific antibodies (total IgG or IgG subtypes IgG1 and IgG2c). In the Sm-cathepsin B/adjuvant formulation group, Sm-cathepsin B specific total IgG titers rose sharply after the first boost at week 3, reaching mean endpoint titers of 40,160 at week 6, and continued to rise until the challenge at week 9 (mean endpoint titers = 122,880) (Fig. 2a). The sera collected from the experimental group revealed robust production of Sm-cathepsin B specific IgG1 that rose sharply after the first boost at week 3. At week 6, the IgG1 mean endpoint titer was 24,960, and it continued to rise until the challenge at week 9 (mean endpoint titer = 48,640) (Fig. 2b). By contrast, Sm-cathepsin B specific IgG2c production was much lower. As seen with the total IgG and IgG1 antibody production patterns, IgG2c titers had the most substantial increase after the first boost at week 3. The mean endpoint titer at week 9 was found to be 1,840 (Fig. 2c).

### Cytokine Production

**Fig. 3:**
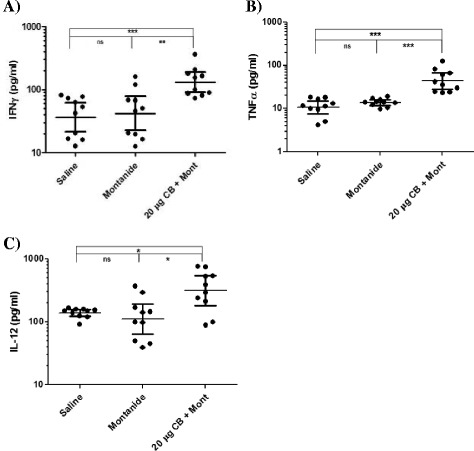
Th1 cytokine secretion levels. IFNγ (*P* = 0.0021) **a**, TNFα (*P* < 0.0001) **b**, and IL-12 (*P* = 0.0185) **c** produced 72 h after stimulating splenocytes ex vivo with recombinant Sm-cathepsin B. Splenocytes were isolated from every C57BL/6 mice belonging to each group: Saline control (saline), Montanide ISA 70VG control (Montanide), and Sm-cathepsin B in the presence of Montanide ISA 720 VG (SmCB + Montanide). Cytokine production was analyzed by QUANSYS multiplex ELISA. Geometric means and 95 % confidence intervals are shown. (ns = not significant, * *P* < 0.05, ** *P* < 0.01, *** *P* < 0.001)

**Fig. 4:**
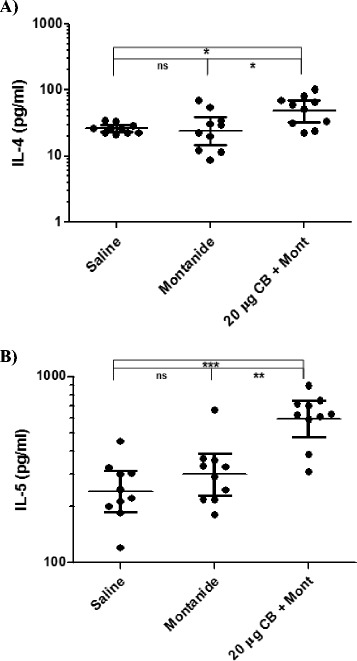
Th2 cytokine secretion levels. IL-4 (*P* = 0.0288) **a** and IL-5 (*P* = 0.0015) **b** produced 72 h after stimulating splenocytes ex vivo with recombinant Sm-cathepsin B. Splenocytes were isolated from every C57BL/6 mice belonging to each group: Saline control (saline), Montanide ISA 70VG control (Montanide), and Sm-cathepsin B in the presence of Montanide ISA 720 VG (SmCB + Montanide). Cytokine production was analyzed by QUANSYS multiplex ELISA. Geometric means and 95 % confidence intervals are shown. (ns = not significant, *P <0.05, **P < 0.01, ***P <0.001)

**Fig. 5:**
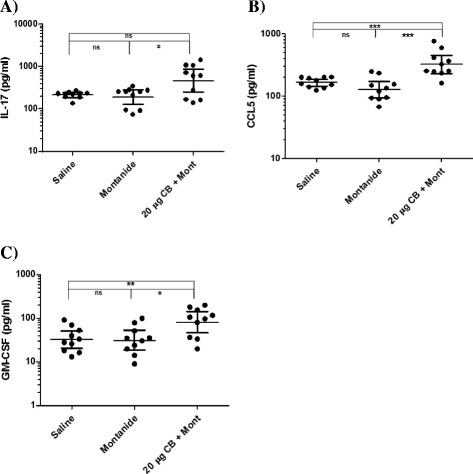
Th17, Chemotactic, and Growth Stimulating Cytokine levels. IL-17 (*P* = 0.0433) **a**, CCL5 (*P* = 0.0002) **b**, and GM-CSF (*P* = 0.0147) **c** produced 72 h after stimulating splenocytes ex vivo with recombinant Sm-cathepsin B. Splenocytes were isolated from every C57BL/6 mice belonging to each group: Saline control (saline), Montanide ISA 70VG control (Montanide), and Sm-cathepsin B in the presence of Montanide ISA 720 VG (SmCB + Montanide). Cytokine production was analyzed by QUANSYS multiplex ELISA. Geometric means and 95 % confidence intervals are shown. (ns = not significant, *P <0.05, **P <0.01, ***P < 0.001)

Analysis of the cytokine panel using QUANSYS multiplex ELISA array revealed no significant differences in cytokine secretion levels between the two control groups (*P* > 0.05). There was an increase in Th1 cytokine production in the group that was immunized with Sm-cathepsin B with Montanide ISA 720 VG compared to the control groups. There was a significant increase in IFNγ secretion levels in the experimental group compared to the Montanide adjuvant control group (*P* = 0.0021) and the saline group (*P* < 0.0001) (Fig. 3a). There were also significant increases in TNFα (*P* < 0.0001) and IL-12 (*P* = 0.0185) levels in the experimental group compared to the adjuvant control group (Fig. 3b and c, respectively). These increases in TNFα and IL-12 levels were also seen when comparing the experimental group to the saline group (*P* < 0.0001 and *P* = 0.0185, respectively). Th2 cytokine production was analyzed as well. There were statistically significant increases in IL-4 levels between the experimental and adjuvant groups (*P* = 0.0288) as well as between the experimental and the saline groups (*P* = 0.0147) (Fig. 4a). IL-5 secretion levels were also significantly increased (*P* = 0.0015) in the group immunized with the Sm-cathepsin B and Montanide ISA 720 VG formulation compared to the adjuvant alone control group (Fig. 4b). This significant increase was also observed when comparing the experimental group to the saline-only group (*P* < 0.0001). IL-17 levels were observed to gain insight into a possible Th17 involvement. Secretion levels of IL-17 were significantly increased in the experimental group compared to the adjuvant control group (*P* = 0.0433). However, this statistically significant increase was not seen when comparing the experimental group to the saline control group (*P* = 0.0892) (Fig. 5a). Other cytokine secretion levels that were significantly increased in the experimental group compared to the adjuvant control group include CCL5 levels (*P* = 0.0002) and GM-CSF levels (*P* = 0.0147) (Fig. 5b and c, respectively). Secretion levels of CCL5 and GM-CSF in the experimental group were significantly higher when compared to the saline group as well (*P* =0.0002 and *P* = 0.0089, respectively).

## Discussion

Due to its essential role in parasite growth and development and its continuous interplay with the host immune system, our group has focused on Sm-cathepsin B as a potential vaccine candidate. During the mid-1990s, the WHO Special Programme for Research and Training in Tropical Diseases (TDR/WHO) planned for the independent testing of various *Schistosoma mansoni* antigens with the hopes of uncovering an optimal vaccine candidate. The committee’s goal was to find an antigen that could consistently induce 40 % protection or better [29]. Recently, our collaborators have demonstrated the inbuilt adjuvant properties of Sm-cathepsin B. Immunizations with unadjuvanted Sm-cathepsin B could decrease both worm and hepatic egg burdens in a mouse model of schistosomiasis by 66 % and 51 %, respectively, when compared to saline control mice [30]. However, the intestinal egg burden was not significantly reduced (24.7 %). The pathological importance of egg burden in the intestines cannot be neglected. The inflammation surrounding eggs trapped in intestinal tissues results in the formation of severe lesions and colonic polyps [31–33]. Furthermore, a decrease in intestinal egg burden could reflect a consequent decrease in transmission by reducing the passage of parasite eggs from the mesenteric veins to the intestinal lumen. The development of an anti-schistosome vaccine needs to target all forms of parasitological burden: worm, hepatic egg, and intestinal egg burdens. The data presented in this manuscript for the formulation of Sm-cathepsin B plus Montanide ISA 720 VG shows comparable protective results for both worm burden; decreased by 60 % compared to adjuvant controls and 66 % compared to saline controls. The reduction in hepatic egg burden was greater in the animals immunized with antigen plus adjuvant (reduction of 62 % compared to adjuvant controls and 69 % compared to saline controls). In this study, we showed that animals immunized with Sm-cathepsin B plus Montanide ISA 720 VG had a significant decrease in intestinal egg burden (56 % compared to the adjuvant control animals and 59 % compared to saline controls). The Montanide-adjuvanted recombinant protein formulation was capable of achieving significant reductions of all parasitological burdens (worms, hepatic eggs, and intestinal eggs). Therefore, using Montanide ISA 720 VG as an adjuvant with the recombinant Sm-cathepsin B improves the protective potential of Sm-cathepsin B.

The *S. mansoni* radiation attenuated (RA) cercariae vaccine has been essential to study immune effector mechanisms associated with protection as well as discovering potential parasite points of vulnerability. The RA vaccine is considered the gold standard for reproducible induced anti-worm immunity. Exposure to the attenuated cercariae can elicit significant protection levels against challenges with normal cercariae. Protection levels have been shown to range from 56 % all the way to 80 % when multiple vaccinations are performed in the mouse model [34–37]. The protection levels attained in this study with Sm-cathepsin B plus Montanide ISA 720 VG, 56-69 %, are beyond the 40 % threshold established by the TDR/WHO, and are comparable to those generated by the RA vaccine.

Several studies have demonstrated that IgG antibodies play an important role in protection against schistosomiasis. The passive transfer of sera from chronically infected or protected animals results in significantly reduced parasite burdens in the recipient animals [38–42]. Studies utilizing the olive baboon and rhesus macaque models have shown that high IgG titers at the time of cercarial challenge correlated with low worm burdens [43, 44]. Furthermore, the worms collected from the macaques, upon perfusion, were physiologically crippled. The compromised structure of these worms is suggested to be a result of antibody-dependant mechanisms where IgG has a blocking or stimulatory effect leading to parasite starvation or exhaustion, respectively [44, 45]. In vitro work using immune sera has also highlighted antibody dependant cell mediated cytotoxicity and antibody dependant complement mediated cytotoxicity as potential effector mechanisms involved in protection against schistosomiasis [41, 46]. Immunizations with the recombinant Sm-cathepsin B in the presence of Montanide ISA 720 VG resulted in the production of elevated antigen-specific IgG titers in the experimental mice (mean endpoint titers at week 9 = 122,880). Immunization with Sm-cathepsin B alone yielded endpoint titers of approximately 3,500 [30] (data not shown) thereby, demonstrating that the addition of the adjuvant Montanide ISA 720 VG increases the production of Sm-cathepsin B specific antibodies by a factor of 35. These values demonstrate this vaccine formulation’s substantial impact on humoral immunity. There were prominent differences when comparing endpoint titers of different IgG subclasses. The adjuvanted recombinant protein formulation elicited a significantly stronger production of the IgG1 subclass (mean endpoint titers at week 9 = 48,640) over IgG2c (mean endpoint titers at week 9 = 1,840). The IgG1 subclass is known to be representative of a T-helper cell 2 (Th2) phenotype as its production is stimulated by IL-4 whereas the IgG2c subclass is a marker for a Th1 [47].

It has long been a consensus that Th1 responses play a key role in protection against schistosomiasis. For instance, peripheral blood mononuclear cells isolated from individuals, who are considered naturally resistant to schistosomiasis, produce high levels of interferon-gamma (IFN-ɣ) when stimulated with schistosome antigen [29, 48]. Moreover, immunological studies involving the RA vaccine have shown that, in protected animals, the migration of the schistosomulae is terminated in the lungs due to the formation of inflammatory foci consisting of monocytes and CD4^+^ T-cells with Th1 characteristics [37, 45, 49]. In the RA model, IL-12 was shown to prime the Th1 response by inducing IFN-ɣ, which then acts in an autocrine manner to amplify the response [37, 45, 49]. The RA vaccine model has also elucidated the important role of TNF-α in protection against schistosomiasis [50]. Both Th1 cytokines IFN-ɣ and TNF-α are secreted by cells in the inflammatory foci, and they are both capable of activating macrophages. Other studies with promising *S. mansoni* vaccine candidates have shown an association between protection against parasite challenge and an increased IFN-ɣ production [51–53]. The formulation of Sm-cathepsin B plus Montanide ISA 720 VG was able to significantly increase the secretion levels of the three Th1 cytokines IFN-ɣ, TNF-α, and IL-12 compared to both the saline and adjuvant controls. However, our experimental formulation was also able to increase the secretion levels of Th2 cytokines; the secretion levels of IL-4 and IL-5 were significantly increased in the experimental animals compared to the controls. Recent studies have discussed the importance of inducing a Th2 response as well to achieve protection against schistosomiasis. Inducing such a response aims to involve eosinophils and basophils in the response to the parasite [30, 54]. The formulation of Sm-cathepsin B in the presence of Montanide ISA 720 VG was able to stimulate both a Th1 (IFN-ɣ, TNF-α, and IL-12) and a Th2 response (IL-4, IL-5, and IgG1). This resulting mixed Th1/Th2 response may explain the higher levels of protection obtained in this study compared to our previous study using a CpG adjuvanted Sm-cathepsin B formulation [24]. The CpG formulation generated a more Th1-biased response. Furthermore, a previous study showed that immunization with Sm-cathepsin B alone leads to increased secretion of Th2-associated cytokines such as IL-4, IL-5, and IL-13, but not Th1-associated cytokines such as IFN-γ thus, creating a Th2-biased response [30]. The Sm-cathepsin B + Montanide ISA 720 VG also significantly increased the secretion levels of IL-17 in the experimental animals compared to the adjuvant control animals. This observation highlights the potential role of Th17 responses in protection against schistosomiasis. Increases in IL-17 levels have been correlated to lower worm burdens [55]. IL-17 may contribute to protection by mediating the recruitment and activation of neutrophils which can impede larval migration via their extracellular traps [54]. Furthermore, this implication of IL-17 was not observed in our previously tested formulation of Sm-cathepsin B + CpG [24] thus, demonstrating the multi-faceted immune involvement mediated by this Montanide adjuvanted formulation.

Levels of both GM-CSF and CCL5 were also significantly increased in the experimental animals compared to the control animals. GM-CSF is involved in macrophage recruitment whereas CCL5 is involved in the recruitment of T cells and granulocytes. Furthermore, CCL5 mediates proliferation and activation of natural killer (NK) cells, which play a role in schistosomiasis protection in elderly populations [56]. Increased secretion levels of CCL5 were also observed with our previous formulation of Sm-cathepsin B + CpG [24]. However, elevated levels of GM-CSF were not observed in our previous formulation thus, representing another immunological difference generated by the formulation containing the Montanide ISA 720 VG adjuvant.

The results obtained from this study are promising and promote further testing of the vaccine candidate Sm-cathepsin B. In the future, the formulation can potentially be tested in a non-human primate model of schistosomiasis. These animals manifest a disease that is similar to that observed in humans, and the data from such a study may be more clinically applicable than data from a mouse study.

## Conclusions

In summary, the data presented in this manuscript demonstrated that immunizations with the formulation of Sm-cathepsin B in the presence of Montanide ISA 720 VG generated protection levels that are comparable to those obtained with the RA vaccine. This formulation elicited a robust production of antigen-specific antibodies and resulted in a favourable mixed Th1/Th2 response. In addition the formulation resulted in the increase of other cytokines such as IL-17 and GM-CSF. The mixed response elicited by the Sm-cathepsin B + Montanide formulation is likely the optimal immune scenario for the host. The essential parasite killing Th1 response was elicited (Th1) as well as the host-beneficial injury repair Th2 response (Th2). The present study along with our previous Sm-cathepsin B study [24] demonstrate the importance of testing different antigen-adjuvant combinations in order to obtain the most protective and immunologically favourable formulation.

## Additional files

Additional file 1: Recombinant Sm-cathepsin B expression. In panel A, the Coomassie stained polyacrylamide gel demonstrates the Sm-cathepsin B representative band at 39 kDa. The same band is also seen upon western blot analysis using anti-His tag antibodies as demonstrated in panel B. (PDF 18 kb) Additional file 2: Parasite burden reduction in experimental mice compared to saline control mice. The experimental mice were immunized with a formulation of 20 μg Sm-cathepsin B + Montanide ISA 720 VG. Statistically significant decreases in all forms of parasite burden (worm, hepatic egg, and intestinal egg numbers) were observed in the experimental mice compared to the saline control mice (mice that were immunized with saline alone). (PDF 131 kb) Additional file 3: Parasite burden reduction in experimental mice compared to adjuvant control mice. The experimental mice were immunized with a formulation of 20 μg Sm-cathepsin B + Montanide ISA 720 VG. Statistically significant reductions in all forms of parasite burden were observed in the experimental mice compared to the adjuvant control mice (mice that were immunized with Montanide ISA 720 VG alone). (PDF 128 kb)
