# Supplementary material for: A vaccine consisting of Schistosoma mansoni cathepsin B formulated in Montanide ISA 720 VG induces high level protection against murine schistosomiasis
Source: BMC Infect Dis. 2016 Mar 5;16:112. doi: 10.1186/s12879-016-1444-z (PMC4779570; doi:10.1186/s12879-016-1444-z)
Supplement: Additional file 1: — Recombinant Sm-cathepsin B expression. In panel A, the Coomassie stained polyacrylamide gel demonstrates the Sm-cathepsin B representative band at 39 kDa. The same band is also seen upon western blot analysis using anti-His tag antibodies as demonstrated in panel B. (PDF 18 kb) [file 12879_2016_1444_MOESM1_ESM.pdf]

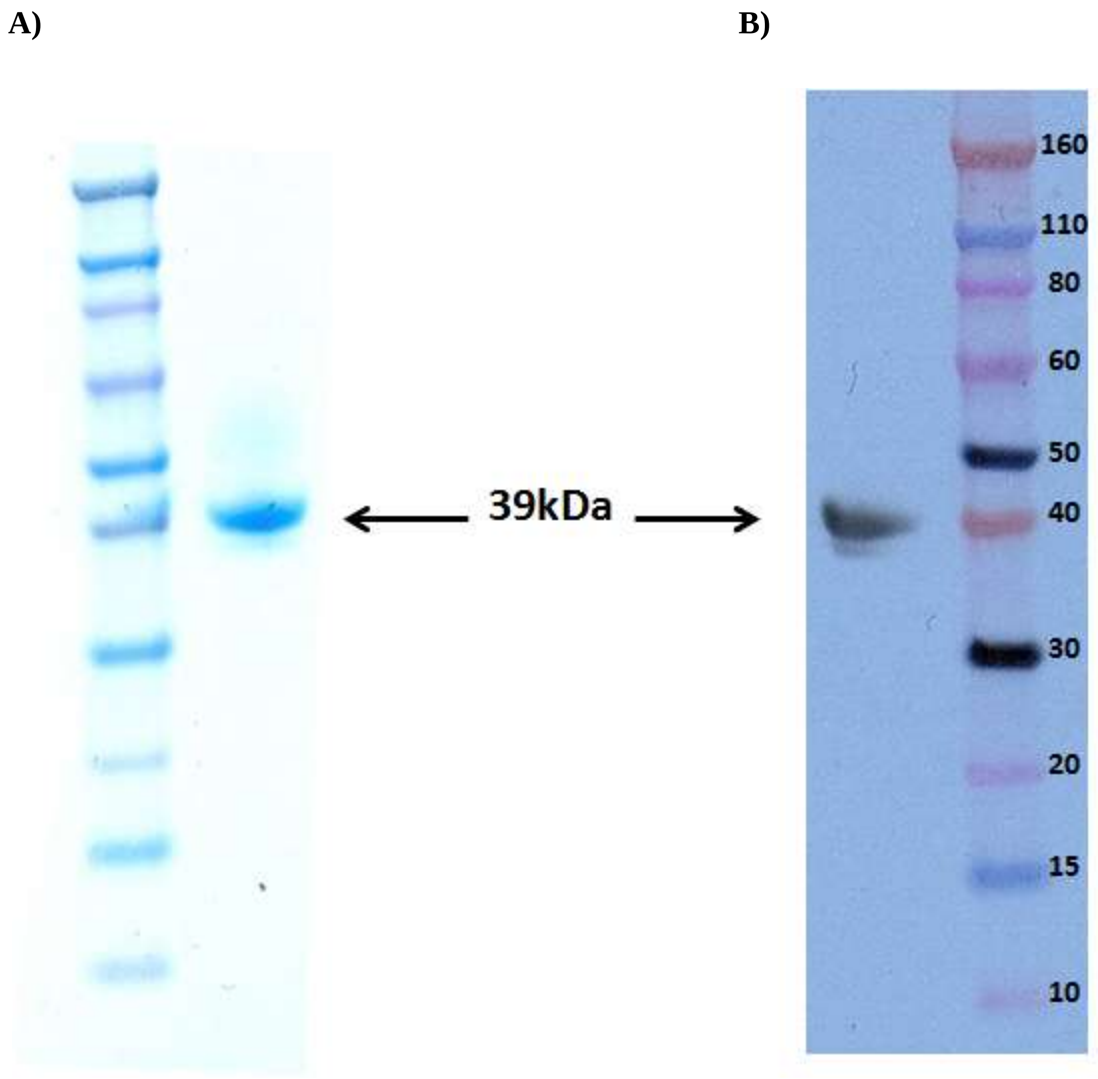

Supplemental Fig 1. Recombinant Sm-Cathepsin B expression in *Pichia pastoris*. The Coomassie stained polyacrylamide gel shows the representative band at 39kDa **(a)**. This same band is also seen upon western blot analysis using anti-His tag antibodies **(b)**.
