# Supplementary material for: A vaccine consisting of Schistosoma mansoni cathepsin B formulated in Montanide ISA 720 VG induces high level protection against murine schistosomiasis
Source: BMC Infect Dis. 2016 Mar 5;16:112. doi: 10.1186/s12879-016-1444-z (PMC4779570; doi:10.1186/s12879-016-1444-z)
Supplement: Additional file 2: — Parasite burden reduction in experimental mice compared to saline control mice. The experimental mice were immunized with a formulation of 20 μg Sm-cathepsin B + Montanide ISA 720 VG. Statistically significant decreases in all forms of parasite burden (worm, hepatic egg, and intestinal egg numbers) were observed in the experimental mice compared to the saline control mice (mice that were immunized with saline alone). (PDF 131 kb) [file 12879_2016_1444_MOESM2_ESM.pdf]

Table 1. Burden reductions observed in mice vaccinated with Sm- Cathepsin B and Montanide ISA 720 VG compared to Saline control mice

**Group**	**Worm Burden (mean ± SE)**	**Percent Reduction (*p*≤0.001)**	**Eggs/g of Liver (mean ± SE)**	**Percent Reduction in experimental group (*p*≤0.001)**	**Eggs/g of Intestine (mean ± SE)**	**Percent Reduction in experimental group (*p*≤0.001)**
Saline (control)	48.8 ± 3.5	-	14791.6 ± 1080.1	-	17800.2 ± 1421.8	-
Sm-Cathepsin B + Montanide ISA 20VG (experimental)	16.4 ± 2.5	66.4%	4578.5 ± 492.9	69.1%	7292.8 ± 1173.1	59.0%

The percent reduction values stated in Table 1 represent the decrease in parasite burden (worms and eggs) observed in the experimental animals when compared to the saline control animals. SE: standard error
